# Perforin Competent CD8 T Cells Are Sufficient to Cause Immune-Mediated Blood-Brain Barrier Disruption

**DOI:** 10.1371/journal.pone.0111401

**Published:** 2014-10-22

**Authors:** Holly L. Johnson, Robin C. Willenbring, Fang Jin, Whitney A. Manhart, Stephanie J. LaFrance, Istvan Pirko, Aaron J. Johnson

**Affiliations:** 1 Department of Neurology, Mayo Clinic, Rochester, Minnesota, United States of America; 2 Department of Immunology, Mayo Clinic, Rochester, Minnesota, United States of America; 3 Neurobiology of Disease Graduate Program, Mayo Graduate School, Rochester, Minnesota, United States of America; 4 Virology and Gene Therapy Graduate Program, Mayo Graduate School, Rochester, Minnesota, United States of America; San Raffaele Scientific Institute, Italy

## Abstract

Numerous neurological disorders are characterized by central nervous system (CNS) vascular permeability. However, the underlying contribution of inflammatory-derived factors leading to pathology associated with blood-brain barrier (BBB) disruption remains poorly understood. In order to address this, we developed an inducible model of BBB disruption using a variation of the Theiler's murine encephalomyelitis virus (TMEV) model of multiple sclerosis. This peptide induced fatal syndrome (PIFS) model is initiated by virus-specific CD8 T cells and results in severe CNS vascular permeability and death in the C57BL/6 mouse strain. While perforin is required for BBB disruption, the cellular source of perforin has remained unidentified. In addition to CD8 T cells, various innate immune cells also express perforin and therefore could also contribute to BBB disruption. To investigate this, we isolated the CD8 T cell as the sole perforin-expressing cell type in the PIFS model through adoptive transfer techniques. We determined that C57BL/6 perforin^−/−^ mice reconstituted with perforin competent CD8 T cells and induced to undergo PIFS exhibited: 1) heightened CNS vascular permeability, 2) increased astrocyte activation as measured by GFAP expression, and 3) loss of linear organization of BBB tight junction proteins claudin-5 and occludin in areas of CNS vascular permeability when compared to mock-treated controls. These results are consistent with the characteristics associated with PIFS in perforin competent mice. Therefore, CD8 T cells are sufficient as a sole perforin-expressing cell type to cause BBB disruption in the PIFS model.

## Introduction

Numerous devastating neurological disorders, including multiple sclerosis, acute hemorrhagic leukoencephalitis (AHLE), dengue hemorrhagic fever, stroke, glioblastoma multiforme, epilepsy, HIV dementia, and cerebral malaria, are characterized by blood-brain barrier (BBB) disruption [1], [2], [3], [4], [5], [6], [7], [8], [9], [10], [11]. Although immune cells have the capacity to initiate CNS vascular permeability, there is relatively little known about how inflammation promotes BBB disruption due to a lack of suitable model systems. This currently undermines the development of therapeutic strategies to ameliorate pathology associated with these disorders. In order to define the mechanisms of BBB disruption, our lab has developed an inducible model of CNS vascular permeability using a variation of the Theiler's murine encephalomyelitis virus (TMEV) model commonly used to study multiple sclerosis [12], [13], [14], [15]. C57BL/6 mice respond to TMEV infection by mounting an antiviral CD8 T cell response that is highly focused on the immunodominant TMEV peptide, VP2_121–130_, presented in the context of the D^b^ class I molecule [16], [17]. However, injection of this immunodominant peptide 7 days post-TMEV infection results in increased astrocyte activation, alteration of BBB tight junctions, severe CNS vascular permeability, and morbidity within 48 hours. This peptide induced fatal syndrome (PIFS) is dependent on virus-specific CD8 T cells and perforin expression [12], [18].

Perforin is a pore forming protein that plays an important role in controlling viral infections and tumors [19]. Perforin has also been shown to play a critical role in an inducible mouse model of seizures, as mice deficient in perforin displayed reduced BBB disruption [6]. When analyzing the effector functions of CD8 T cells in our PIFS model system, we found that perforin, but not Fas ligand, was required for pathology associated with PIFS to develop. In these experiments, we determined C57BL/6 perforin^−/−^ mice are resistant to PIFS and are devoid of CNS vascular permeability as measured by magnetic resonance imaging (MRI) analysis and leakage of FITC-albumin into the CNS parenchyma. Astrocyte activation, as measured by glial fibrillary acidic protein (GFAP) expression, was also found to be dependent on perforin expression in the PIFS model. Events indicative of BBB disruption are dependent on perforin expression [18]. However, the cellular source of perforin required for promoting BBB disruption is unknown. In addition to CD8 T cells, natural killer (NK) cells and γδ T cells express perforin and have been shown to use perforin-mediated cytotoxicity during viral infections [20], [21], [22]. Neutrophils have also recently been shown to express perforin to regulate immune responses in allergic contact dermatitis [23]. Therefore, while we have previously demonstrated that both CD8 T cells and perforin are critical factors causing BBB disruption, it remained unknown the extent other perforin-expressing immune cell types assisted in the development of PIFS.

Since PIFS is initiated by class I-restricted virus antigen, we hypothesized that CD8 T cells directly use perforin to cause BBB disruption independent of other immune cell types. We tested this hypothesis using adoptive transfer techniques to isolate the CD8 T cell as the sole perforin-expressing cell type in the PIFS model. After reconstituting perforin^−/−^ mice with perforin competent CD8 T cells, mice were intracranially infected with TMEV and administered either PIFS-inducing VP2_121–130_ peptide or mock E7 peptide 7 days post-infection. Mice were then evaluated for activation of astrocytes, disruption of the tight junction organization, and CNS vascular permeability in order to determine whether perforin competent CD8 T cells alone are sufficient to cause BBB disruption.

## Materials and Methods

### Animals and ethics statement

C57BL/6 perforin deficient male mice (strain #002407) were obtained from Jackson laboratories (Bar Harbor, ME) at 5 weeks of age. C57BL/6-Tg(UBC-GFP) (strain #004353) male mice were bred in-house at Mayo Clinic. C57BL/6 Ly5.1 male mice were provided by Dr. Adam Schrum, Mayo Clinic. All experiments were approved by the Institutional Animal Care and Use Committee of Mayo Clinic (protocol number A57913).

### Adoptive transfer

**Figure 1:**
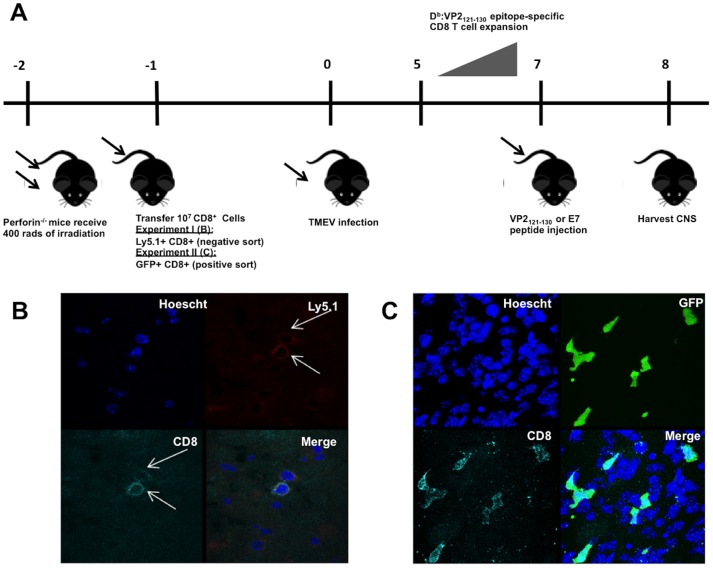
Adoptive transfer of CD8+ cells successfully migrate to brain post induction of PIFS. (A) Schematic illustrating the adoptive transfer of sorted perforin competent CD8+ cells into perforin^−/−^ mice followed by induction of PIFS. C57BL/6 perforin^−/−^ mice were irradiated with 400 rads of irradiation and then intravenously injected with 10^7^ GFP+ CD8+ splenocytes (n = 10) or 10^7^ Ly5.1+ CD8+ splenocytes (n = 10). Mice were intracranially infected with TMEV on the following day. PIFS-inducing VP2_121–130_ peptide or mock E7 control peptide were intravenously administered on day 7, during the peak of CD8 T cell expansion. MRI analysis was performed on the following day to visualize the extent of CNS vascular permeability and then the CNS was harvested for additional assays. Both negative (Experiment I) and positive (Experiment II) sort experiments yielded high purity of CD8+ T cell transfer. (B) Representative confocal microscopic images illustrating co-localization of Ly5.1 and CD8 protein (Experiment I). CNS-infiltrating Ly5.1+ cells colocalized with CD8+ cells 85.7% of the time as measured by confocal analysis. (C) Confocal microscopy showing of representative brain tissue slice showing successful transfer of GFP+ CD8+ T cells (Experiment II). Purity of transfer was analyzed via flow cytometry, 98.0%+− 0.5% of cells that were CD8 positive and GFP positive.

Spleens of GFP+ or Ly5.1+ mice were removed and strained through a nylon mesh 100 µm filter. CD8+ cells were purified from the resulting lymphocyte population using MACS LS cell purification columns (Miltenyi Biotec, Auburn, CA) according to the manufacturer's protocol. Positive cell sorting was used to obtain GFP+ CD8+ cells and negative cell sorting was used to obtain Ly5.1+ CD8+ cells. Both methods resulted in ∼90% purity as determined by flow cytometric analysis (data not shown). C57BL/6 perforin^−/−^ mice were irradiated with 400 rads of irradiation, given one day to recover, and then intravenously injected with 10^7^ Ly5.1+ CD8+ or GFP+ CD8+ splenocytes (Figure 1A).

### Induction of CNS vascular permeability using the PIFS model

CNS vascular permeability was induced using the PIFS model as previously described [12], [18], [24]. Briefly, all mice were intracranially infected with 2 x 10^6^ PFU Daniel's strain of TMEV. Seven days post-TMEV infection, during the peak of CD8 T cell expansion in the brain, mice were intravenously administered 0.1 mg VP2_121–130_ (FHAGSLLVFM) peptide or mock control E7 (RAHYNIVTF) peptide (Genescript) (Figure 1A).

### Injection of rhodamine-dextran or FITC-albumin to assess permeability

Mice were given an intravenous injection of 10 mg rhodamine B isothiocyanate-dextran (Sigma #R9379) or 10 mg FITC-albumin (Sigma #A9771) 23 hours after VP2_121–130_ or E7 peptide administration. Brains were harvested at 24 hours.

### Flow Cytometry

Brain-infiltrating lymphocytes were isolated from the left hemisphere through collagenase digestion and a percoll gradient as previously described [25]. Isolated brain lymphocytes were then incubated with anti-CD45 PerCP (BD Biosciences, 557235) and anti-CD8 PE-Cy7 (BD Biosciences, 552877) for 40 minutes. Cells were washed twice with FACS buffer and then fixed in 4% paraformaldehyde and 1X PBS. Samples were analyzed on a BD LSRII flow cytometer (BD Biosciences) using FACS Diva software (BD Biosciences).

### Rhodamine-dextran permeability assay

Mice were intravenously injected with 10 mg rhodamine B isothiocyanate-dextran (Sigma #R9379) 23 hours after VP2_121–130_ or E7 peptide administration. Brains were harvested one hour later and frozen on aluminum foil on dry ice. The right hemisphere was homogenized with radioimmunoprecipitation assay (RIPA) buffer [10 mmol/L Tris, 140 mmol/L NaCl, 1% Triton X-100, 1% Na dexycholate, 0.1% SDS and protease inhibitor cocktail (Pierce #78410) pH 7.5] and centrifuged for 10 minutes at 10,000 rpm. Protein concentration was assessed using the BCA protein assay (Pierce #23223) and then samples were normalized for protein content. Homogenates were read on a fluorescent plate reader at 540 nm excitation and 625 nm emission to detect rhodamine-dextran leakage into the brain. Data were collected using SpectraMax software (Molecular Devices).

### Confocal Microscopy

Fresh frozen coronal slices from mice injected with FITC-albumin, were cut on a cryostat and placed onto positively charged slides. Slides were washed twice with PBS and then fixed in 3% paraformaldehyde for 15 minutes. After the fixation step, slides were rinsed three times in PBS and then incubated for 1 hour in 5% normal goat serum + 0.5% Igepal CA-630 (Sigma I3021) in PBS. To determine the percentage of CNS-infiltrating Ly5.1+ CD8 T cells, slides were incubated with the following primary antibodies at a concentration of 1:100 overnight at room temperature: anti-mouse CD45.1 PE (eBioscience, 12-0453-82) and rat anti-mouse CD8 (AbD Serotec, MCA2694). The following secondary antibodies were used at a concentration of 1:250 for 1 hour at room temperature: AlexaFluor 532 goat anti-mouse IgG (Invitrogen, A-11002) and AlexaFluor 647 goat anti-rat IgG (Invitrogen, A21247). To visualize the tight junction organization, slides were incubated with the following primary antibodies at a concentration of 1:100 overnight at room temperature: mouse anti-claudin-5 (Invitrogen, 35–2500) and rabbit anti-occludin (Invitrogen, 71–1500). The following secondary antibodies were then added at a concentration of 1:250 for 1 hour at room temperature: AlexaFluor 532 goat anti-mouse IgG (Invitrogen, A-11002) and AlexaFluor647 goat anti-rabbit IgG (Invitrogen, A-21244). To assess astrocyte activation, slides were incubated with mouse anti-GFAP-Cy3 (Sigma, 106K4775) at a concentration of 1:500 overnight at room temperature. All slides were rinsed 5 times in PBS before and after incubation with secondary antibodies. This was followed by Hoechst staining at a concentration of 1:500 in PBS for 5 minutes. Slides were then rinsed 5 times in PBS, dried, and covered with Vectashield medium (Vector lab, H-1000). Images were acquired using a Leica (Germany) DM 2500 confocal microscope equipped with a 63x oil immersion objective (numerical aperture 1.30). All images were collected at room temperature using Type F immersion liquid (Leica Microsystems) and analyzed using LAS AF stimulator AF 6000 acquisition software. All images shown in manuscript are at 63x magnification.

### MRI Acquisition

A Bruker Avance II 7 Tesla vertical bore small animal MRI system (Bruker Biospin) was used for image acquisition to evaluate CNS vascular permeability as previously described [9], [26]. Briefly, inhalation anesthesia was induced and maintained using 3–4% isoflurane. An MRI compatible vital sign monitoring system (Model 1030, SA Instruments, Inc., Stony Brook NY) was used to monitor respiratory rate during acquisition. Gadolinium was intraperitoneally administered to mice using weight based dosing of 100 mg/kg. After a standard delay of 15 minutes, a volume acquisition T1-weighted spin echo sequence was used (TR-150 ms, TE = 8 ms, FOV: 32 mm x 19.2 mm x 19.2 mm, Matrix: 160 x 96 x 96, number of averages = 1) to obtain T1-weighted images.

### Image Analysis

3D volumetric analysis was performed as previously described [25], [27], [28], [29]. Briefly, Analyze 11.0 software (Biomedical Imaging Resource, Mayo Clinic) was used to quantify the 3D volume of CNS vascular permeability. The 3D volume extractor tool was used to extract brains from gadolinium-enhanced T1-weighted images. The 3D ROI tool was then used to define areas of gadolinium leakage using semi-automated methods. The volume of these areas, which corresponds to the volume of gadolinium leakage, was calculated using the 3D sampling tool. 3D object rendering using the volume rendering tool was performed to visualize the identified volumes of contrast enhancement. All 3D volumes were standardized to the mean of control (mice receiving E7 peptide on day 7) within an experiment and plotted as individual mice, shown as Ratio of Gadolinium Volume.

### Statistical Analysis

Mean and standard error values were calculated using SigmaStat software (SYSTAT Software Inc). GraphPad Prism Software was used to construct graphs with standard error bars. A Student's t-test was performed using SigmaStat to evaluate CNS vascular permeability, as detected by leakage of rhodamine-dextran into the CNS, and FITC intensity from confocal microscopic images. A Welch test was used to evaluate 3D volumetric analysis of vascular leak visible on T1-weighted MRI scans.

## Results

### Adoptively transferred perforin competent CD8 T cells induce CNS vascular permeability

#### Model and adoptive transfer purity

CD8 T cells that recognize the immunodominant D^b^:VP2_121–130_ epitope initiate BBB disruption [12]. However, the extent CD8 T cell expressed perforin contributes to CNS vascular permeability remains unknown. Therefore, we developed an adoptive transfer approach to isolate the CD8 T cell as the sole perforin-expressing cell type (Figure 1A). CD8+ cells were purified from the spleens of Ly5.1+ perforin+/+ mice using negative cell sort (Experiment I), or GFP+ perforin^+/+^ mice using positive cell sorting (Experiment II) resulting in ∼90% purity as determined by flow cytometric analysis (data not shown). Recipient C57BL/6 perforin^−/−^ mice were irradiated with 400 rads of irradiation, given one day to recover, and then intravenously injected with 10^7^ sorted CD8+ splenocytes. The following day mice were intracranially infected with TMEV. PIFS-inducing VP2_121–130_ peptide or mock E7 control peptide were intravenously administered on day 7, during the peak of CD8 T cell expansion (Figure 1). Gadolinium-enhanced T1-weighted MRI was performed on the following day to visualize CNS vascular permeability. Immediately following MRI, the CNS was harvested to be further analyzed by confocal microscopy, flow cytometry and CNS vascular permeability was measured by fluorescent molecule diffusion across the BBB.

To determine the percentage of CNS-infiltrating Ly5.1+ CD8 T cells post-induction of PIFS, tissue slices were stained with antibody to Ly5.1 and CD8 and observed under a confocal microscope. Comparable numbers of CNS-infiltrating Ly5.1+ CD8 T cells were observed in both VP2_121–130_ peptide (n = 6) and E7 peptide-treated (n = 4) groups, with the average being 85.7% (Figure 1B, data not shown). Flow cytometric analysis was used to determine the percentage of CNS-infiltrating GFP+ CD8 T cells post-induction of PIFS. Isolated CNS-infiltrating lymphocytes were stained with antibody to CD45 and CD8. The CD45^hi^ population was then gated and analyzed for the percentage of GFP+ CD8 T cells. Comparable numbers of CNS-infiltrating CD8+ cells as a percentage of GFP+ cells were observed in both VP2_121–130_ peptide (n = 6) and E7 peptide-treated (n = 4) groups, with the average being 98.0%±0.5% (data not shown). Furthermore, tissue slices were stained with antibody to CD8 and GFP and observed using confocal microscopy and defined to be high purity (Figure 1C).

#### CNS permeability measured by small animal MRI and fluorescent molecule diffusion

**Figure 2:**
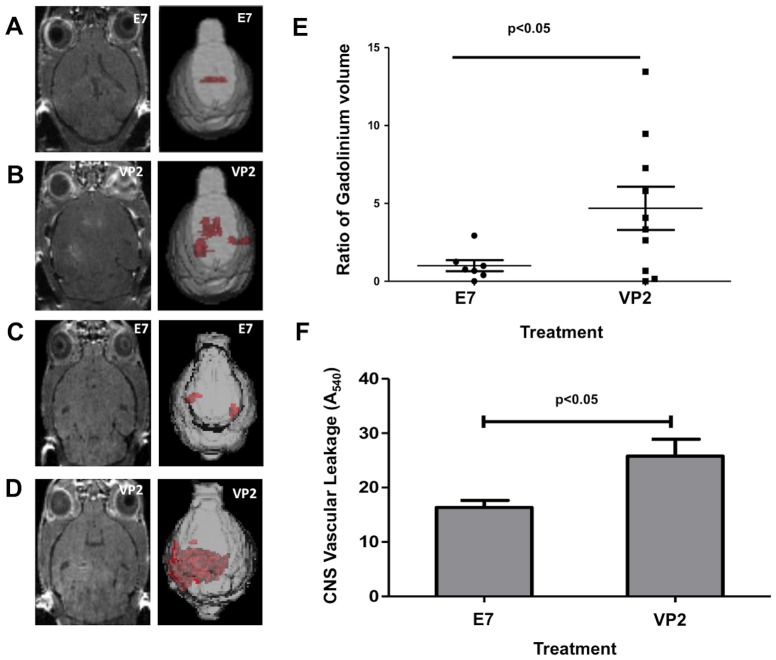
CD8 T cells as sole perforin wielding source are sufficient to induce CNS vascular permeability. The extent of CNS vascular permeability is illustrated by raw image and 3D transparency rendering of gadolinium-enhancing areas from T1-weighted MRI scans in mice administered (A,C) mock E7 control peptide or (B,D) VP2_121–130_ peptide. MRI images are separated to show both methods of CD8+ T cell selection (A,B) negative sort or (C,D) positive sort to isolate and transfer CD8+ cells. (E) Quantification of the 3D volume of vascular leakage from all mice (both negative and positive sort) revealed that VP2_121–130_ peptide-treated mice having CD8 T cells as the sole perforin-expressing cell type have a significantly more amount of gadolinium leakage (p<0.05) (n = **5,9** per group). (F) A significant increase in CNS vascular permeability detectable by intravenously injected rhodamine dextran accumulation in brain homogenates was observed in VP2_121–130_ peptide-treated animals (n = 6) compared to mock E7 peptide-treated animals (n = 4) (p<0.05). Results are depicted as mean ± SEM.

In order to quantitatively evaluate the full volume of CNS vascular leak in animals undergoing PIFS, where CD8 T cells were the sole perforin-expressing cell type, we performed volumetric analysis of 3D gadolinium-enhanced T1-weighted MRI scans using Analyze 11.0 software developed by Mayo Clinic's Biomedical Imaging Resource [30], [31]. Quantification of the 3D volume of gadolinium leakage from vasculature revealed that VP2_121–130_ peptide-treated mice reconstituted with perforin competent CD8 T cells had increased CNS vascular permeability (Figure 2 B,D) when compared to mock E7 peptide-treated controls (Figure 2 A,C) (p<0.05, Figure 2E). CD8+ cells were selected using a negative (Figure 2 A,B) or positive (Figure 2 C,D) sort method. Importantly, an additional VP2_121–130_ peptide-treated mouse, not included in this data set, was moribund and unable to be scanned. Post-mortem analysis revealed that this mouse had the highest transfer of CD8 T cells (data not shown).

Another group of animals was injected with rhodamine-dextran 23 hours post-administration of mock E7 peptide or VP2_121–130_ peptide to induce PIFS. Rhodamine-dextran was allowed to circulate for one hour. Brains were then harvested and the right hemisphere was used to assess CNS vascular permeability. In concordance with the T1-weighted MRI analysis, a significant increase in vascular permeability was observed in the VP2_121–130_ peptide-treated group (n = 6) compared to the E7 peptide-treated group (n = 4), as detected by leakage of rhodamine-dextran into the CNS using a fluorescent plate reader (A_540_) (p<0.05) (Figure 2F).

### Increased astrocyte GFAP expression in perforin^−/−^ mice reconstituted with perforin competent CD8 T cells

**Figure 3:**
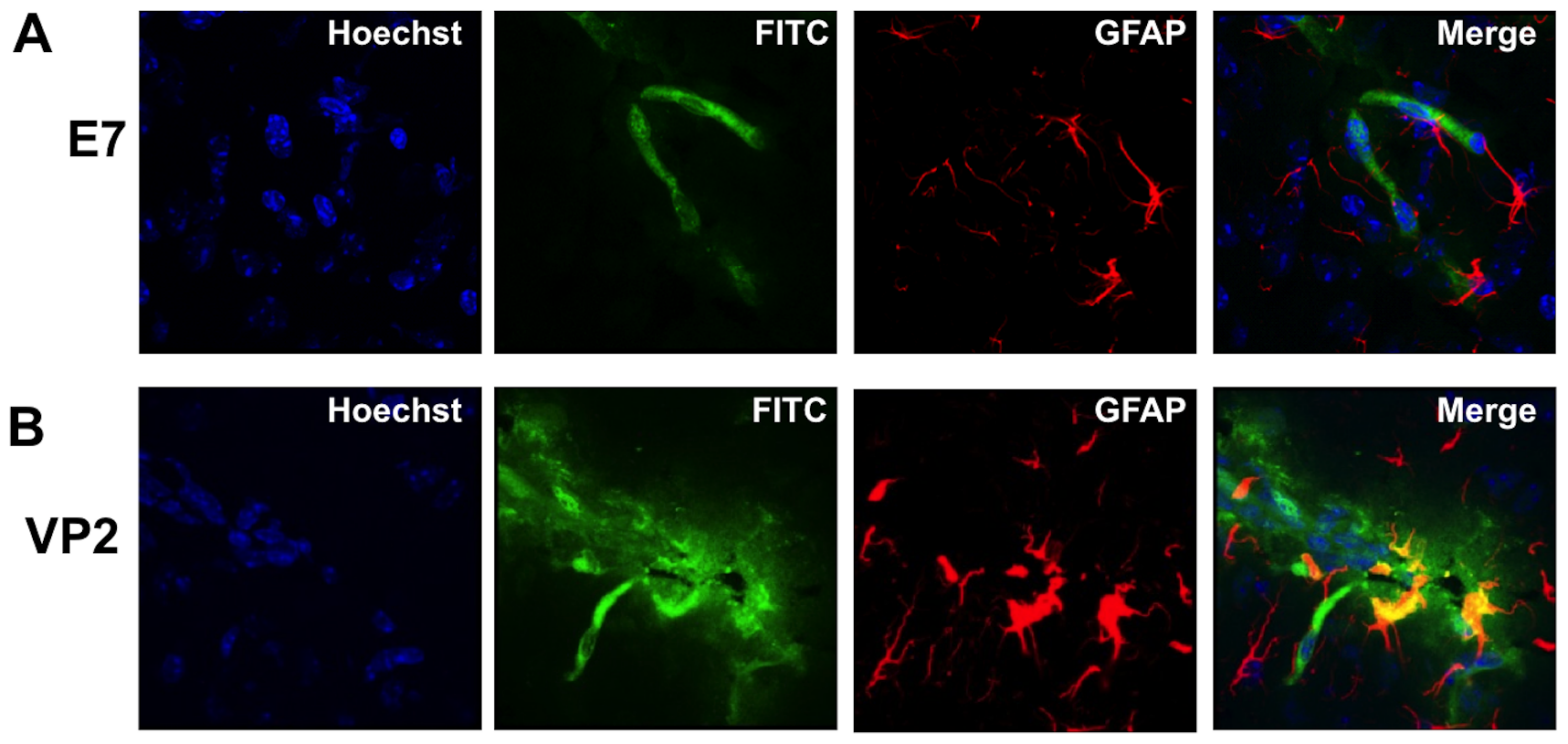
Increased astrocyte activation colocalizes with CNS vascular permeability post-induction of PIFS in perforin^−/−^ mice reconstituted with perforin competent CD8 T cells. Representative confocal microscopic images illustrating astrocyte expression of GFAP in (A) E7 peptide-treated (n = 4) and (B) VP2_121–130_ peptide-treated (n = 6) perforin^−/−^ mice reconstituted with perforin competent CD8 T cells. Mice treated with (B) VP2_121–130_ peptide displayed increased astrocyte activation, as measured by heightened GFAP expression, in areas of CNS vascular permeability when compared to (A) mock E7 peptide-treated negative control mice. CNS permeability is determined by leakage of intravenously administered FITC-albumin.

Activation of astrocytes, as measured by GFAP expression, co-localizes with CNS vascular permeability in C57BL/6 mice induced to undergo PIFS [18]. To determine the extent astrocytes were activated in perforin^−/−^ mice reconstituted with perforin competent CD8 T cells, we stained for astrocyte expression of GFAP and performed confocal microscopy. We found that mice treated with VP2_121–130_ peptide displayed an increase in astrocyte size consistent with astrogliosis (Figure 3B), when compared to E7 peptide-treated mice (Figure 3A). We also observed colocalization of increased astrocyte activation with CNS vascular permeability. This is consistent with previous studies in which PIFS was evaluated in perforin competent wild-type C57BL/6 mice [18]. In this study, in accordance with previous studies, there is a clear increase in astrocyte activation in areas with vascular leak as compared to unaffected tissue (Figure 3B).

### Disorganized tight junction proteins colocalize with increased CNS vascular permeability in perforin^−/−^ mice reconstituted with perforin competent CD8 T cells

**Figure 4:**
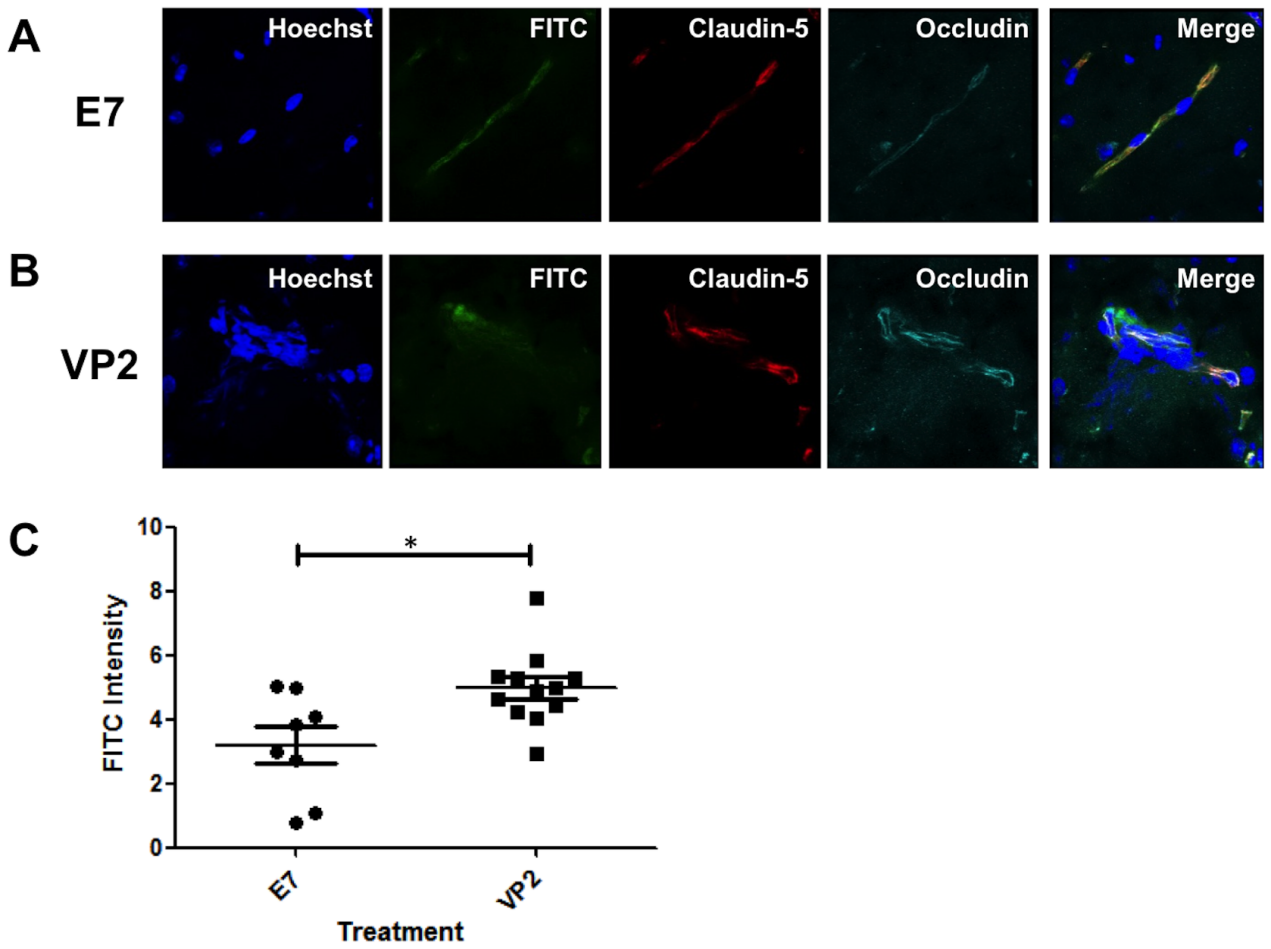
Disorganization of tight junction proteins and increased CNS vascular permeability can be induced in perforin^−/−^ mice reconstituted with perforin competent CD8 T cells. Confocal microscopic images from a representative (A) mock E7 peptide-treated C57BL/6 perforin^−/−^ mouse reconstituted with perforin competent CD8 T cells depict a preservation of vascular integrity and intact tight junction proteins claudin-5 and occludin. (B) VP2_121–130_ peptide-treated C57BL/6 perforin^−/−^ mice reconstituted with perforin competent CD8 T cells display degradation of tight junction proteins claudin-5 and occludin in areas of CNS vascular permeability as seen through FITC-albumin leakage. (C) FITC intensity in brain tissue from two representative confocal microscopic images of each animal was quantified using ImageJ software. VP2_121–130_ peptide-treated mice (n = 6) displayed a significant higher intensity of FITC-albumin in brain parenchyma when compared to E7 peptide-treated mice (n = 4) (p<0.05).

The increased vascular permeability in perforin^−/−^ mice reconstituted with perforin competent CD8 T cells prompted additional analysis of focal areas of CNS vascular permeability and the state of the BBB tight junction proteins. We determined that mock E7 peptide-treated C57BL/6 perforin^−/−^ mice reconstituted with perforin competent CD8 T cells display preservation of vascular integrity, as shown by lack of FITC-albumin leakage into the brain parenchyma from the brain vasculature, and linear organization of BBB tight junction proteins claudin-5 and occludin (Figure 4A). However, C57BL/6 perforin^−/−^ mice reconstituted with perforin competent CD8 T cells and administered VP2_121–130_ peptide to induce PIFS presented with a loss of linear organization of tight junction proteins claudin-5 and occluding in the microvessels. Disorganization of BBB tight junctions colocalizes with FITC-albumin leakage from brain vasculature (Figure 4B). FITC intensity from confocal microscopic images was then quantified using ImageJ software in brain tissue slices. Two random representative images from each animal were analyzed. FITC intensity values were normalized through subtraction of background values. We determined using this method that VP2_121–130_ peptide-treated mice (n = 6) displayed a significant higher intensity of FITC-albumin when compared to E7 peptide-treated mice (n = 4) (p<0.05) (Figure 4C). The colocalization of disorganized tight junction proteins with CNS vascular permeability in this experiment is consistent with previous studies performed in perforin competent wild-type C57BL/6 mice [18], [25]. Vascular leak and disorganization of BBB tight junction proteins is evident with transfer of perforin competent CD8 T cells into a perforin-/- mouse and induced to undergo PIFS.

## Discussion

Investigating the underlying molecular mechanisms leading to pathology associated with BBB disruption is of critical importance for the development of therapeutic approaches to treat diseases characterized by CNS vascular permeability. Using the PIFS model system, our lab has previously demonstrated critical roles for CD8 T cells and perforin in promoting activation of astrocytes, loss of linear organization of the tight junction, and extensive CNS vascular permeability. We have also shown that inhibiting the functions of these critical players improves pathology and survival [12], [18]. Furthermore, we have demonstrated that other molecular players implicated in causing BBB disruption, such as GR-1+ neutrophils, CD4 T cells, TNF-α, IFN-γ, LTβR, and IL-1, do not contribute to lethality in CD8 T cell-initiated BBB disruption [16], [25]. However, whether CD8 T cells are the cellular source of perforin required for promoting BBB disruption was not known.

In addition to CD8 T cells, NK cells, γδ T cells, and neutrophils also use perforin as an effector mechanism. [20], [21], [22], [23], [32], [33], [34]. Mice deficient in NK cells displayed a higher degree of inflammation and demyelination after induction of experimental autoimmune encephalomyelitis (EAE) [32], [33], [34]. Depletion of NK cells has also been shown to result in more severe encephalitis in mice post-TMEV infection [21]. Additionally, mice deficient in γδ T cells exhibited increased viral load and were more susceptible to West Nile Virus infection. These mice also displayed decreased levels of intracellular perforin in splenocytes post-infection with West Nile Virus and had a lower cytotoxicity compared to wild-type mice [22]. Furthermore, neutrophils have been shown to use perforin to regulate CD8 T cell infiltration into the skin to mediate allergic contact dermatitis [23]. This raises the question as to whether other molecular players such as NK cells, γδ T cells, and neutrophils contribute to perforin-dependent CD8 T cell-mediated BBB disruption during neuroinflammation.

In this study, we tested our hypothesis that CD8 T cells employ perforin to cause BBB disruption independent of other immune cell types. We developed an adoptive transfer approach to isolate perforin competent CD8 T cells and determine if they are sufficient to cause activation of astrocytes, disorganization of BBB tight junction proteins, and CNS vascular permeability. Using this adoptive transfer approach, we found that perforin^−/−^ mice reconstituted with perforin competent CD8 T cells and induced to undergo PIFS exhibited a significant increase in CNS vascular permeability, measured by fluorescent molecule diffusion across the BBB, compared to mock E7 peptide-treated controls. This prompted a more comprehensive analysis using small animal MRI and confocal microscopy. Quantification of the 3D volume of gadolinium leakage from vasculature visible on T1-weighted MRI scans illustrated that PIFS-inducing VP2_121–130_ peptide-treated mice reconstituted with CD8 T cells as the sole perforin-expressing cell type displayed significant increase in CNS vascular permeability when compared to mock E7 peptide-treated controls. This analysis showed that perforin competent CD8 T cells are sufficient to induce CNS vascular permeability.

We then used confocal microscopy to analyze BBB disruption in more detail by examining astrocyte activation, the organization of BBB tight junction proteins claudin-5 and occludin, and focal areas of vascular permeability. This analysis demonstrated that perforin competent CD8 T cells are sufficient to cause increased astrocyte activation and disorganization of the tight junctions in areas of CNS vascular permeability.

Studies using the EAE model have also put forward a role for astrocytes in causing disorganization of BBB tight junction proteins and ensuing CNS vascular permeability. These studies have proposed a mechanism in which CD4 T cells induce astrocytes to release vascular endothelial growth factor (VEGF), resulting in BBB disruption [35]. However, in contrast to EAE, CD4 T cells do not contribute to lethality in the PIFS model [36]. Furthermore, while we have also demonstrated a critical role for VEGF in promoting BBB disruption, we found that neurons were the major source of VEGF expression during CNS vascular permeability [24]. However, since we have observed increased astrocyte activation colocalizing with CNS vascular permeability, there is still a potential role for astrocytes in the PIFS model. It is possible that increased astrocyte activation causes a permeable state or is a consequence of BBB disruption. This will be a topic of further investigation.

This study has expanded our knowledge on the mechanism of BBB disruption by illustrating that perforin competent CD8 T cells are sufficient to cause traits associated with immune-mediated BBB disruption. It remains necessary to investigate the functional mechanism used by perforin to cause BBB disruption. In experimental cerebral malaria, granzyme B has been defined as a critical factor in promoting fatal cerebral pathology [37]. The extent granzyme B contributes to BBB disruption in PIFS remains to be defined. Furthermore, the complete mechanism of how perforin enables delivery of granzymes to cause apoptosis of target cells also remains to be defined. It was originally thought that perforin created pores on target cells to allow entry of granzymes, resulting in cell death [38]. Recently it has been suggested that the perforin pore actively, and selectively, passes cationic molecules, such as granzyme B, more efficiently that anionic and neutral molecules [39]. Alternative models propose that granzymes are endocytosed independent of perforin. This is followed by perforin acting on the endosomal membrane to form pores, causing the release of granzymes, which in turn promotes apoptosis of target cells [40], [41]. A recent proposed mechanism involves a combination of these two pathways. In this third model, perforin forms pores in the plasma membrane of the target cell. This results in an influx of Ca^2+^ that triggers repair of the plasma membrane. Granzymes and perforin are then endocytosed together into large endosomes, and perforin acts on the endosomal membrane to release granzymes into the cytosol. However, the mechanism of how perforin causes release of granzymes from the endosome remains unclear [42].

Perforin is commonly defined as providing cytolytic activity against target cells [38]. Recently new, non-classical, mechanisms have been put forward that perforin can function in non-cytolytic pathways. Perforin has been identified, along with granzyme, as having a noncytolytic role in controlling the reactivation of HSV-1 neuronal infections without inducing cytotoxicity [43]. Additionally, in mouse model of obesity related insulin resistance and visceral adipose tissue (VAT), a lack of perforin showed reduced insulin sensitivity and changed the inflammation status within the VAT lesions, suggesting an immunoregulatory role for perforin in this disease state. Importantly, in this study, the formation of crown like structures, indicative of adipocyte death, was not changed in mice lacking perforin, further emphasizing a noncytolytic role of perforin [44]. Other noncytolytic roles for perforin extend to contributing to CD8 T cell activation during *arenavirus* infection and regulating antigen presentation of dendritic cells (DCs) [45], [46]. Previous work, in our lab, shows caspase-3 cleavage is not present until after BBB disruption has already occurred [18]. Given these new roles for perforin and lack of evidence for apoptosis, it is possible that during BBB disruption, perforin is acting in a non-classical mechanism. However, the exact mechanism by which perforin wielding CD8 T cells can induce BBB disruption is yet to be defined.

The contribution of molecules delivered by perforin beyond granzyme B also needs to be evaluated. For example, orphan granzymes have recently been proposed to play a role in pathogen clearance and cell-mediated death [47]. Nevertheless, based on the findings put forward in this manuscript, investigating mechanisms of perforin-dependent cytotoxicity are important to the development of novel therapeutic strategies to ameliorate pathology associated with BBB disruption in several devastating neurological disorders. The demonstration that CD8 T cells can serve as a sole source of perforin to induce BBB disruption will greatly aid in this process.
